# Supplementary figures and images for: Single-cell transcription profiles in Bloom syndrome patients link BLM deficiency with altered condensin complex expression signatures
Source: Hum Mol Genet. 2022 Jan 31;31(13):2185–93. doi: 10.1093/hmg/ddab373 (PMC9262399; doi:10.1093/hmg/ddab373)

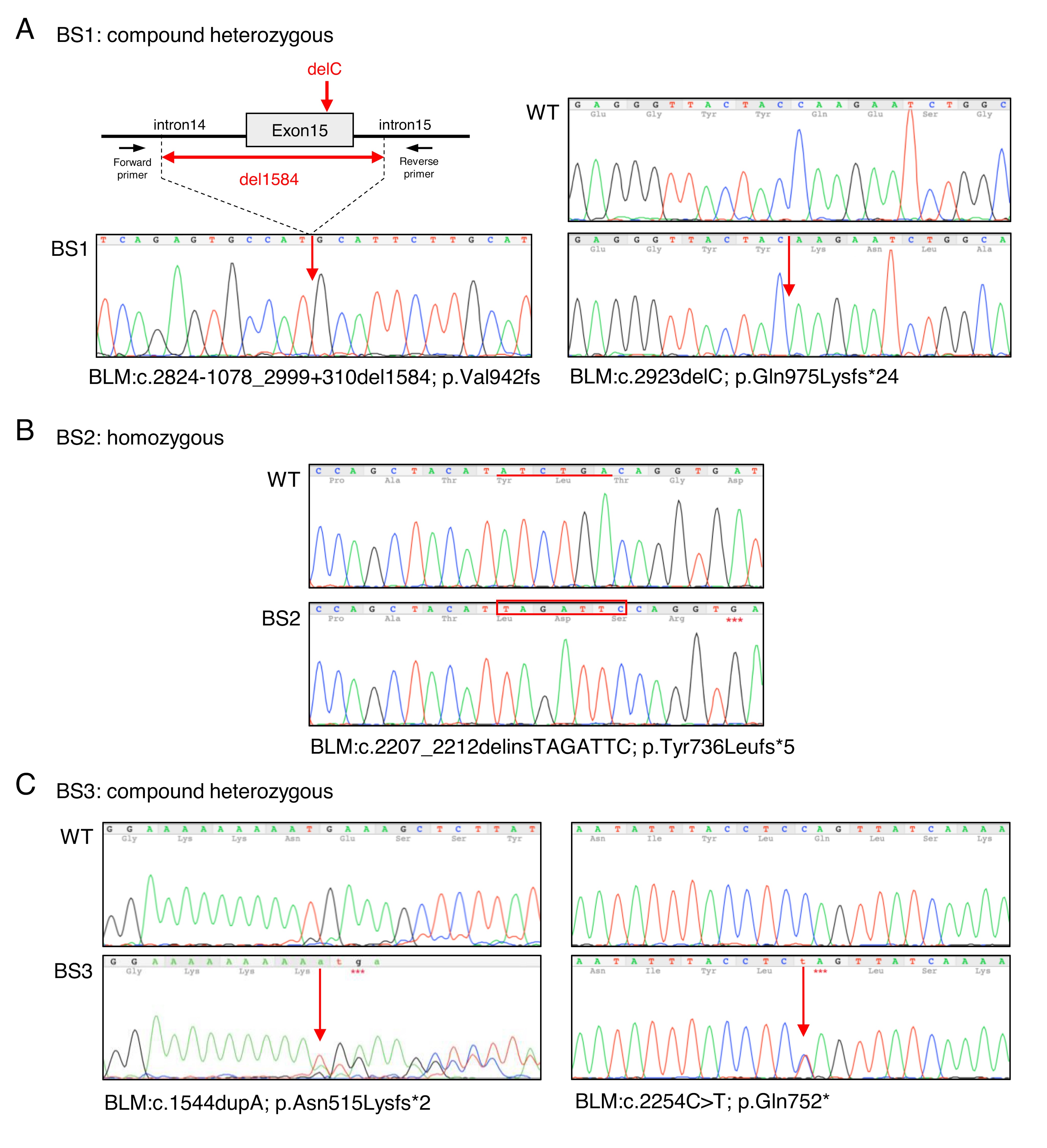

Supplement: HMG-2021-CE-00720_Suppl_Fig_S1_ddab373 [file hmg-2021-ce-00720_suppl_fig_s1_ddab373.jpeg]

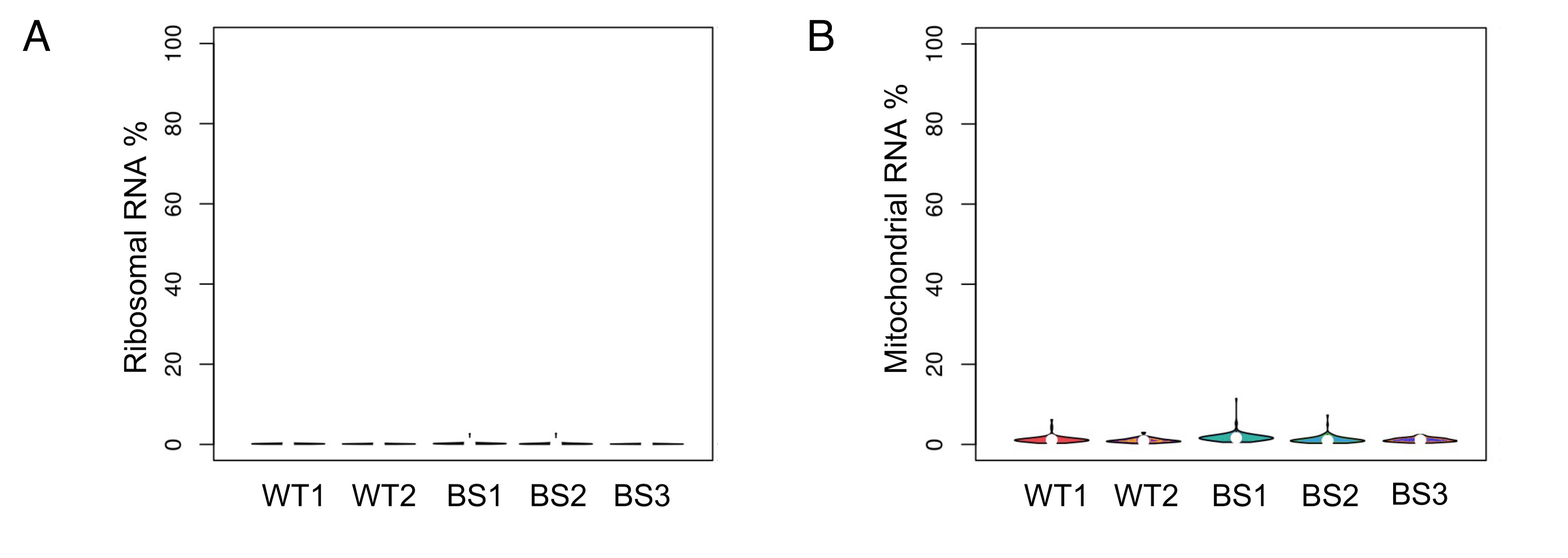

Supplement: HMG-2021-CE-00720_Suppl_Fig_S2_ddab373 [file hmg-2021-ce-00720_suppl_fig_s2_ddab373.jpeg]

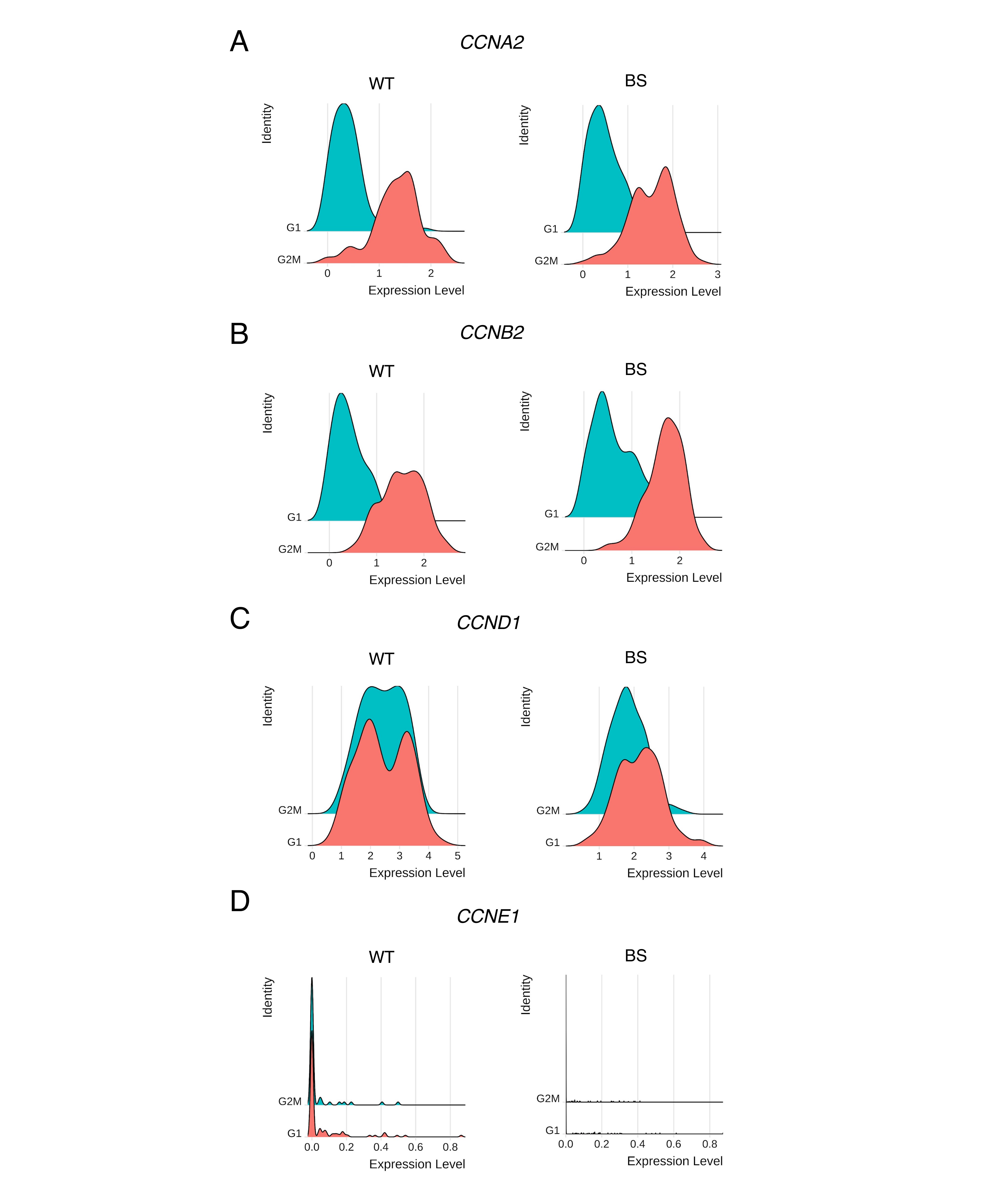

Supplement: HMG-2021-CE-00720_Suppl_Fig_S3_ddab373 [file hmg-2021-ce-00720_suppl_fig_s3_ddab373.jpeg]

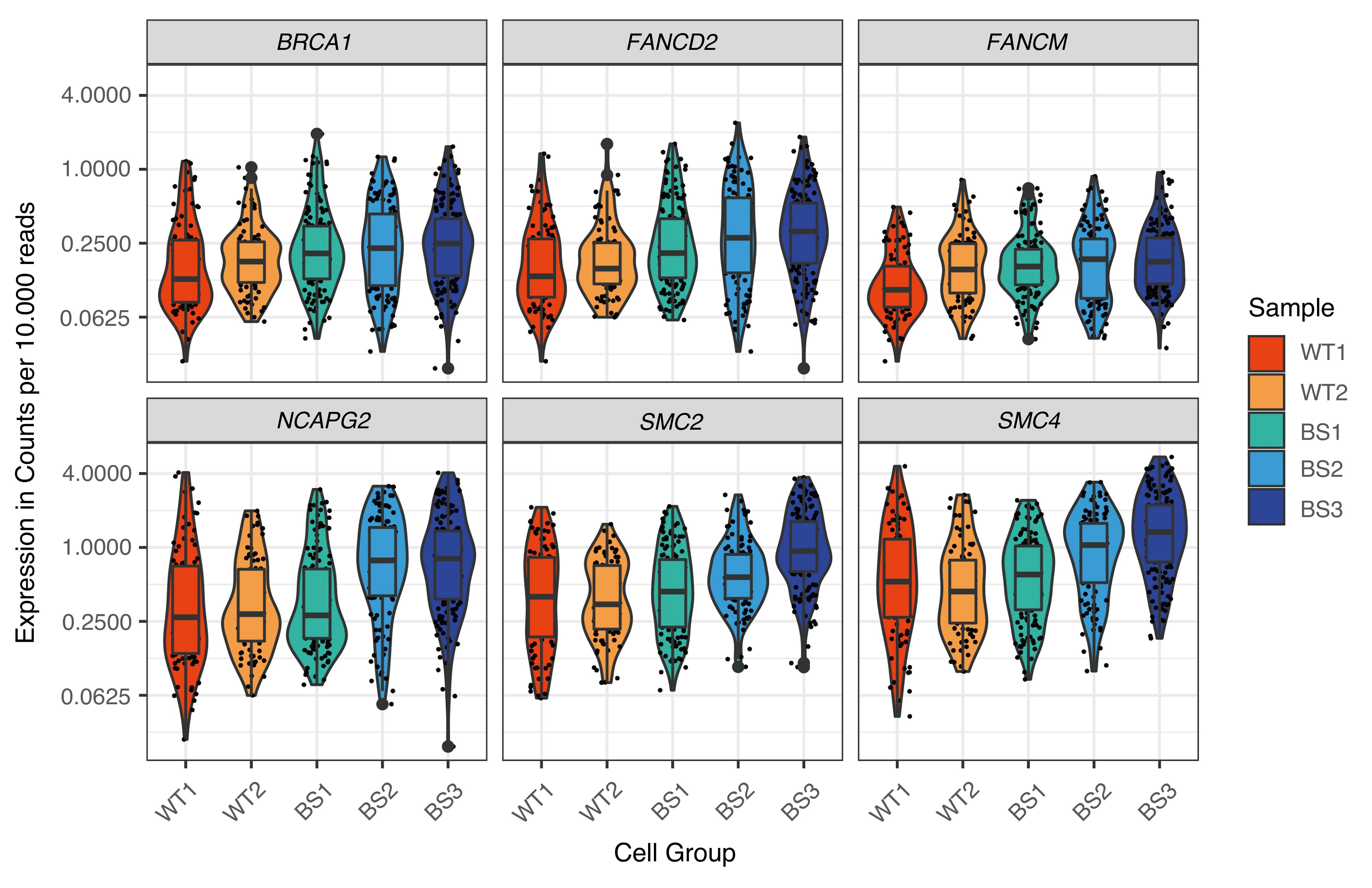

Supplement: HMG-2021-CE-00720_Suppl_Fig_S4_ddab373 [file hmg-2021-ce-00720_suppl_fig_s4_ddab373.jpeg]

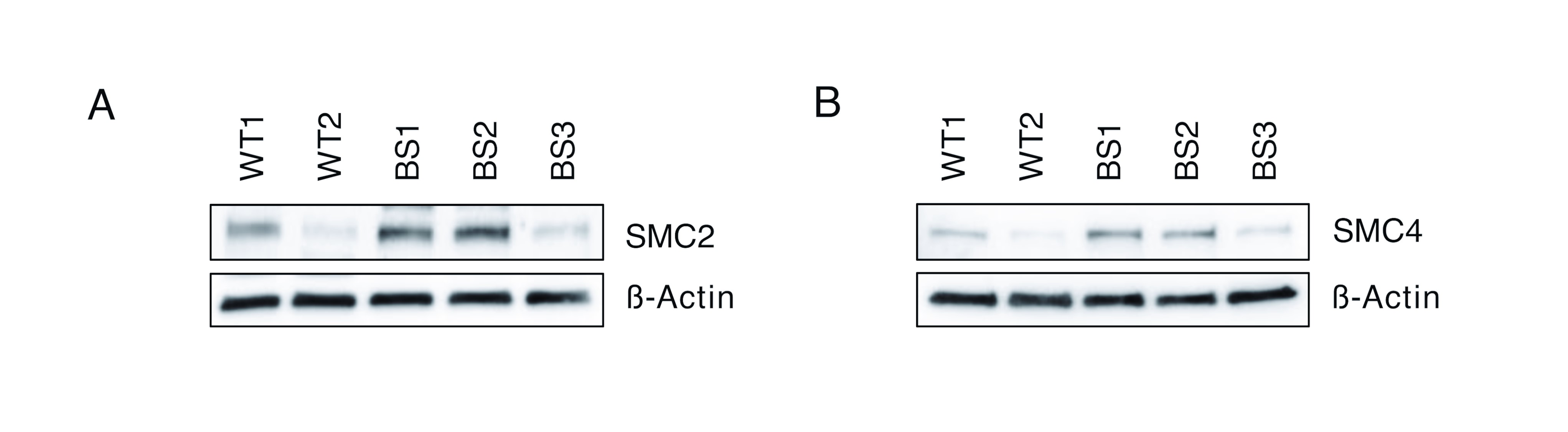

Supplement: HMG-2021-CE-00720_Suppl_Fig_S5_ddab373 [file hmg-2021-ce-00720_suppl_fig_s5_ddab373.jpeg]
