# Supplementary material for: Single-cell transcription profiles in Bloom syndrome patients link BLM deficiency with altered condensin complex expression signatures
Source: Hum Mol Genet. 2022 Jan 31;31(13):2185–93. doi: 10.1093/hmg/ddab373 (PMC9262399; doi:10.1093/hmg/ddab373)
Supplement: HMG-2021-CE-00720_Supplementary_text_final_ddab373 [file hmg-2021-ce-00720_supplementary_text_final_ddab373.docx]

**Supplementary Tables**

Supplementary Table 1. Cell line details used in the study

Supplementary Table 2: Differentially expressed genes in BS cells in comparison to WT

Supplementary Table 3: Genes that are included in Figure 3 and the corresponding values

**Legends to Supplementary Figures**

**Figure S1.** *BLM* gene mutations of the cell lines used in this study were confirmed by Sanger sequencing. (A) BS1 cell line was compound heterozygous, (B) BS2 cell line was homozygous, and (C) BS3 cell line was compound heterozygous for the corresponding loss-of-function mutations in the *BLM* gene. Red arrows indicate the positions of the mutations on the alleles of Bloom syndrome patient cell lines on part (A) and (C). Red underlined sequence shows the deleted sequence on wild-type whereas the sequence marked with red rectangle shows the inserted sequence on the BS patient cell line on part (B). Nomenclature is according to *BLM* transcript NM_000057.4.

**Figure S2.** Additional quality control elements support the robustness of the single-cell transcriptome sequencing technique. (A) Ribosomal RNA percentage shows almost no ribosomal RNA detected in the raw data of all samples. (B) Mitochondrial RNA percentage shows less than 10% of overall mitochondrial gene expression among all genes for all samples. Plots were obtained using CogentAP pipeline (Cogent NGS Analysis Pipeline, Takara Bio).

**Figure S3.** No difference in the expression of cell cycle marker genes between WT and BS cells was observed. All fibroblast cell samples analyzed presented similar proportions on cell cycle phases, e.g., G1 and G2/M. Ridge plots show expression levels of specific cyclins such as (A) *CCNA2,* (B) *CCNB2,* (C) *CCND1*, and (D) *CCNE1* in wild type (WT) and Bloom syndrome (BS) single-cell groups. Briefly, a score for each cell was determined based on the expression of a setup of G2/M- and S phase-specific markers. Then, the expression levels of different cyclins were compared between two cell groups (WT and BS). Expression level is given on the x-axis and the distribution of the number of cells per cell-cycle phase is shown on the y-axis (G1 or G2/M). Higher peaks represent more cells having the corresponding expression level for the related gene. Plots were generated as described in (1) using the vignette of Seurat (2).

**Figure S4**. Gene expression levels (counts per 10,000) of each individual sample (two WT and three BS samples) are shown for different genes on a single-cell level. From left to right; on the upper row *BRCA1*, *FANCD2*, and *FANCM*, from the Fanconi anemia pathway; and on the bottom row *NCAPG2,* *SMC2,* and *SMC4* presenting genes encoding condensin complex I/II are shown. The expression level differences of shown genes were significant after multiple test adjustments and the adjusted p-values are given in Supplementary Table 2. For the corresponding genes, the median expression levels were higher in BS than in WT. Visualization was done via R-package ggplot2 (3).

Figure S5. Western blots show the existence of main members of condensin I and II complexes on the cell lysates of fibroblast cell lines used in this study. (A) *SMC2,* (B) *SMC4* genes were transcribed and translated on all of the cell lysates. Higher expression levels of *SMC2* and *SMC4* genes in the BS cell lines (BS1, BS2, and BS3) can be observed in protein level in comparison to control cell lines (WT1 and WT2). The experiments were done in triplicates and beta-Actin was used as a control.

**References**

1. Hussmann, J.A., Ling, J., Ravisankar, P., Yan, J., Cirincione, A., Xu, A., Simpson, D., Yang, D., Bothmer, A., Cotta-Ramusino, C., *et al.* (2021) Mapping the genetic landscape of DNA double-strand break repair. *Cell*, **184**, 5653-5669.e25.

2. Hao, Y., Hao, S., Andersen-Nissen, E., Mauck, W.M., Zheng, S., Butler, A., Lee, M.J., Wilk, A.J., Darby, C., Zager, M., *et al.* (2021) Integrated analysis of multimodal single-cell data. *Cell*, **184**, 3573-3587.e29.

3. Wickham, H., Navarro, D. and Pedersen, T.L. (2016) ggplot2, Elegant Graphics for Data Analysis. *ggplot2, Elegant Graphics for Data Analysis*; 3rd ed.; Springer, New York, NY, New York, NY, (2016) .
